# Application of Language Models for the Analysis of Adverse Drug Events in Pharmaceutical Research and Development: Scoping Review

**DOI:** 10.2196/77732

**Published:** 2026-06-16

**Authors:** Oren Schreier, Anthony Yazdani, Ioannis Galdadas, Ryme Kabak, Francesco Luigi Gervasio, Gang Mu, Douglas Teodoro

**Affiliations:** 1 Department of Radiology and Medical Informatics, Faculty of Medicine, University of Geneva, Chemin des Mines 9, Geneva, 1202, Switzerland, 41 0223790225; 2 School of Pharmaceutical Sciences, University of Geneva, Geneva, Switzerland; 3 Institute of Pharmaceutical Sciences of Western Switzerland (ISPSO), University of Geneva, Geneva, Switzerland; 4 Swiss Institute of Bioinformatics, University of Geneva, Geneva, Switzerland; 5 Johnson & Johnson World Headquarters, Bridgewater, NJ, United States; 6 Department of Chemistry, University College London, London, England, United Kingdom; 7 Cilag GmbH International, Zug, Switzerland

**Keywords:** adverse drug events, artificial intelligence, language models, drug development, pharmacovigilance, pharmaceutical research, risk assessment, information extraction

## Abstract

**Background:**

Adverse drug events (ADEs) remain a critical safety issue in pharmaceutical research and development (Pharma R&D), necessitating robust methods for early detection and surveillance. Language models (LMs) are increasingly used in ADE analysis, addressing safety challenges during drug development and postmarket surveillance. Language modeling approaches, ranging from static embeddings to large language models (LLMs), capitalize on diverse data sources, such as clinical trial datasets, electronic health records, and social media posts, to predict ADEs, analyze real-world evidence, and improve drug screening and pharmacovigilance systems.

**Objective:**

This scoping review aims to map the application of LMs for the analysis of ADEs across the Pharma R&D lifecycle.

**Methods:**

Following the PRISMA-ScR (Preferred Reporting Items for Systematic Reviews and Meta-Analyses Extension for Scoping Reviews) guidelines, we searched PubMed, Web of Science, and Google Scholar for relevant papers published between January 2015 and October 2025.

**Results:**

This review identified 49 relevant papers. Overall, LM applications in Pharma R&D safety analysis are concentrated in 2 distinct phases: ADE prediction during the premarket phase (n=16) and ADE detection in postmarket surveillance (n=33).

**Conclusions:**

While some models demonstrate high predictive performance, persistent challenges, including data heterogeneity and limited external validation, hinder widespread adoption. Despite these barriers, discriminative and generative LMs have the potential to transform drug safety across the pre- and postapproval phases, especially when integrated with real-world pharmacovigilance frameworks.

## Introduction

Adverse drug events (ADEs), injuries caused by the use or misuse of medications, pose a major challenge throughout the entire drug development lifecycle [1-3]. It is estimated that over 30% of drug candidates are discarded owing to toxicity, even after they are launched on the market [4]. High rates of ADEs have significant consequences for patient safety and health care systems. For example, in the postmarket setting, the prevalence of adverse drug reactions among hospitalized older adults is 22%; yet, 60% are preventable cases largely driven by predictable factors such as polypharmacy and complex comorbidities [5]. Catastrophic drug safety failures like the thalidomide disaster of 1961, which caused severe birth defects in thousands of infants, underscored the need for rigorous pharmacovigilance. These concerns highlight the importance of early detection and prediction of ADEs across the entire drug development lifecycle, from preclinical testing to phase IV clinical trials.

The identification of new ADEs caused by a drug product is one of the key activities in the pharmaceutical industry to ensure the safety profile of a drug product. However, assessing the safety of a drug well before it reaches the market is not always straightforward. Drug candidates that appear safe in preclinical stages can exhibit toxicity in clinical phases, leading to high failure rates. One contributing factor to this attrition is the discrepancy between animal models used in preclinical screenings and human biology, where preclinical safety data fail to predict human reactions [6-8]. Consequently, ADEs, including treatment-related fatalities, can emerge even during controlled clinical trials [9]. Since premarket testing cannot always guarantee safety, rigorous phase IV surveillance remains essential to identify risks once the drug enters the broader population. Postmarket monitoring relies heavily on spontaneous reporting systems. However, these systems are known to substantially undercount true ADEs [10], notably due to limited clinician time and the complexity of reporting workflows [11].

In the modern era, the volume and variety of drug safety data have grown significantly, encompassing not only structured sources, such as trial registries and spontaneous reporting systems, but also diverse real-world data streams, including electronic health records (EHRs) and social media data [12,13]. This abundance of data has outpaced traditional surveillance approaches and created a need for automated methods to monitor drug safety signals. Machine learning (ML) has the potential to assist with signal detection and supplement traditional pharmacovigilance surveillance methods [14] due to its capacity for multimodal and large-scale data processing. Among the different ML approaches, language models (LMs) have emerged as a versatile technology for addressing such safety challenges, mostly given their ability to process extremely diverse data, where safety risks can be found in unstructured human language text, such as physician notes, biomedical literature, and social media posts, or encoded in chemical language, where molecular structures are represented as text sequences. Consequently, the field of LMs for drug safety has witnessed a methodological evolution over the last decade. While earlier approaches relied on static representations, recent years have seen a shift toward contextualized architectures and generative Large Language Models (LLMs) [15]. These advancements have enabled diverse analyses, ranging from extracting ADE mentions in patient forums to predicting complex toxicity endpoints based solely on molecular formulations.

Several reviews concerning the use of artificial intelligence (AI) to analyze ADEs are already available, although these sources either miss current developments in LMs or only focus on a specific aspect. In particular, the application of AI to ADE prediction has already been the subject of 3 scoping and 2 systematic reviews between 2022 and 2025 (Table 1). Syrowatka et al [16], in their scoping review, discuss a series of use cases to identify the most promising areas in which AI can be used to reduce the frequency of ADEs, but exclude studies that included postmarket surveillance. Yang and Kar [17] cover a much broader area of the different aspects that contribute to the resulting ADEs, with a strong focus on toxicity prediction, and discuss how AI and ML techniques can be applied in this area. The work of Denck et al [18] highlights the ability of AI or ML to analyze large datasets and identify complex patterns in observational health data, thereby improving drug safety and pharmacovigilance. It also discusses limitations, such as the need for high-quality data and the challenges of model interpretability and generalizability. Although the scoping review by Hu et al [19] focuses on AI methods that use EHR to predict ADEs, the use of only 10 studies limits the generalizability of their observations. Finally, Teodoro et al [20] review diverse AI algorithms for safety, efficacy, and operational risks in clinical trials, noting the recent emergence of LLMs. However, their broad focus on general ML and multiple risk categories limits their specific analysis of LMs applied to safety in the pharmaceutical research and development (Pharma R&D) lifecycle.

Distinct from existing reviews, our scoping review focuses on the application of LMs across the entire Pharma R&D pipeline, from preclinical discovery to postmarket surveillance. Our goal is to present a comprehensive layout of how LMs serve as a unifying technology, bridging the methodological gap between premarket toxicity prediction and postmarket surveillance. By covering the time frame from 2015 to October 2025, we capture the technological shift from static embeddings (eg, word2vec) to the emergence of LLMs. Through this analysis, we aim to map recent methodological developments and key trends, but also highlight future research directions based on the outstanding challenges of current approaches. To this end, we address the following research questions:

- RQ1: For which organs and toxicity endpoints are LMs used for in safety analysis?
- RQ2: What types of LMs have been used to analyze safety risks in drug design and development?
- RQ3: What are the data sources and metrics used for training and evaluating LM methods for safety assessment in Pharma R&D?
- RQ4: What are the current limitations of LM approaches for AI-based ADE analysis?

**Table 1.** Overview of existing literature.

Authors	Journal	Year	Scope
Syrowatka et al [16]	*The Lancet Digital Health*	2022	ScR^a^: ML^b^ and AI^c^ techniques for pharmacovigilance with a focus on detecting ADEs^d^
Yang and Kar [17]	*Artificial Intelligence Chemistry*	2023	SR^e^: AI and ML methods and databases for early detection of ADEs and toxicity
Denck et al [18]	*Drug Discovery Today*	2023	SR: ML approaches for the prediction of ADEs from observational health data
Hu et al [19]	*Frontiers in Pharmacology*	2024	ScR: application of ML algorithms in predicting specific ADEs using EHR^f^ data
Teodoro et al [20]	*npj Digital Medicine*	2025	ScR: a scoping review of AI applications in clinical trial risk assessment

a ScR*:* scoping review.

b ML: machine learning.

c AI: artificial intelligence.

d ADE: adverse drug event.

e SR: systematic review.

f EHR: electronic health record.

## Methods

Our systematic search covers peer-reviewed studies published in English between January 1, 2015, and October 15, 2025. Our selection process followed the PRISMA-ScR (Preferred Reporting Items for Systematic Reviews and Meta-Analyses Extension for Scoping Reviews) guidelines (Checklist 1).

### Search Strategy and Study Selection

In the search phase, we used 3 major databases: PubMed, Web of Science, and Google Scholar. We queried databases for potentially relevant records using a broad range of keywords stratified into five groups: (1) Pharma R&D, (2) drug-related terms, (3) ADE-related terms, (4) the type of algorithm that was used, and (5) the task that the algorithm was supposed to perform. We combined the keywords within each group using the OR operator, and all groups were combined using the AND operator. To search for papers, we applied the default settings of the respective databases using the title and abstract fields. Table 2 contains the search keywords used in the process.

**Table 2.** Keyword groups for the search strategy^a^.

Group	Category	Keywords
1	Pharma R&D^b^-related keywords	“clinical research” OR “clinical trials” OR “pharmaceutical research” OR “pharmacological research” OR “pharmaceutical development” OR “drug design” OR “drug development” OR “pharmacovigilance” OR “event detection”
2	Drug-related keywords	“drug” OR “compound” OR “substance”
3	Adverse drug event–related keywords	“adverse drug reaction” OR “adverse drug event” OR “toxicity”
4	Machine learning keywords	“artificial intelligence” OR “language model” OR “fuzzy” OR “rule-based” OR “machine learning” OR “support vector machine” OR “decision tree” OR “neural network” OR “deep learning” OR “text mining” OR “natural language processing”
5	Task-related keywords	“predict” OR “extract” OR “detect” OR “classify”

a The final query included all the described groups.

b Pharma R&D: pharmaceutical research and development.

To ensure relevance, we applied specific inclusion and exclusion criteria, as reported in Textbox 1, namely papers written in English, with ADEs as the main topic, involving mammalian species, and published in peer-reviewed journals or conference proceedings between January 1, 2015, and October 15, 2025. To focus on the recent trend in the AI field, we excluded papers that did not use LMs in their modeling approach.

Textbox 1. **Criteria for including and excluding studies.**

**Inclusion criteria**

- Adverse drug events are the main topic of the paper
- Basic research
- Peer-reviewed papers published in journals and conferences
- English language
- Publication date between January 1, 2015, and October 15, 2025
- All papers retrieved in PubMed and Web of Science, and the top 188 papers in Google Scholar

**Exclusion criteria**

- Risk factor analyses
- Nonlanguage modeling algorithms
- Nonpharmacological treatment
- Adverse drug events in nonmammalian species
- Clinical application (as opposed to pharmaceutical research and development)
- Cross-drug interaction (polypharmacy)

### Dataset Screening and Annotation

Three researchers (OS, AY, and DT) independently screened the titles and abstracts. The resulting set was cross-checked by IG and RK. Then, OS, AY, IG, RK, and DT read the full texts independently and extracted item information using a standard spreadsheet created before the analysis in line with the research questions. Any differences in including or excluding full-text studies were resolved during a consensus meeting. The final dataset was based on the CHARMS (Critical Appraisal and Data Extraction for Systematic Reviews of Prediction Modeling Studies) checklist and includes the publication date, whether the study was published in a journal or a conference, country of the corresponding author, source of data used, task formulation, toxicity endpoints that were predicted, affected organ, metrics of performance evaluation, the algorithm used, the nature of the algorithm used, the type of LM, the features that were used for the modeling, and the drug design and development stage.

### Data Analysis

We analyzed the data using Microsoft Excel for Mac (Microsoft Office 365, version 16.69). We used descriptive statistics like frequencies and ranges and presented the data graphically and in tabular format, as needed.

## Results

### Overview

**Figure 1.**
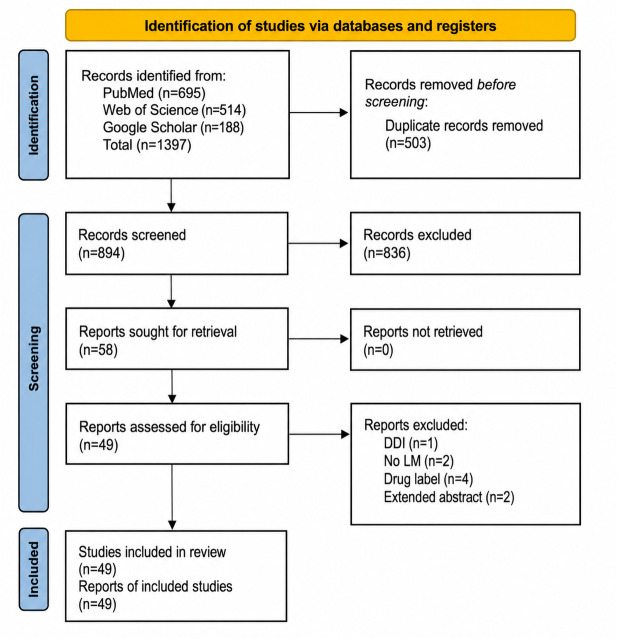
PRISMA flowchart describing the different literature sources used, and the selection process followed to filter down the relevant sources that were used in the end (adapted from Page et al [21], which is published under Creative Commons Attribution 4.0 International License [22]). Only studies whose methodology involved LMs were included. DDI: drug-drug interaction; LM: language model; PRISMA: Preferred Reporting Items for Systematic Reviews and Meta-Analyses.

The search query resulted in 1397 records from PubMed (n=695), Web of Science (n=514), and Google Scholar (n=188), which, after removing duplicates (n=503), and performing a “title & abstract” screening (n=894; n=836 records excluded), and a full-text eligibility assessment (n=58; n=9 records excluded), were narrowed down to 49 records included for analysis. Our study selection flowchart is shown in Figure 1.

**Figure 2.**
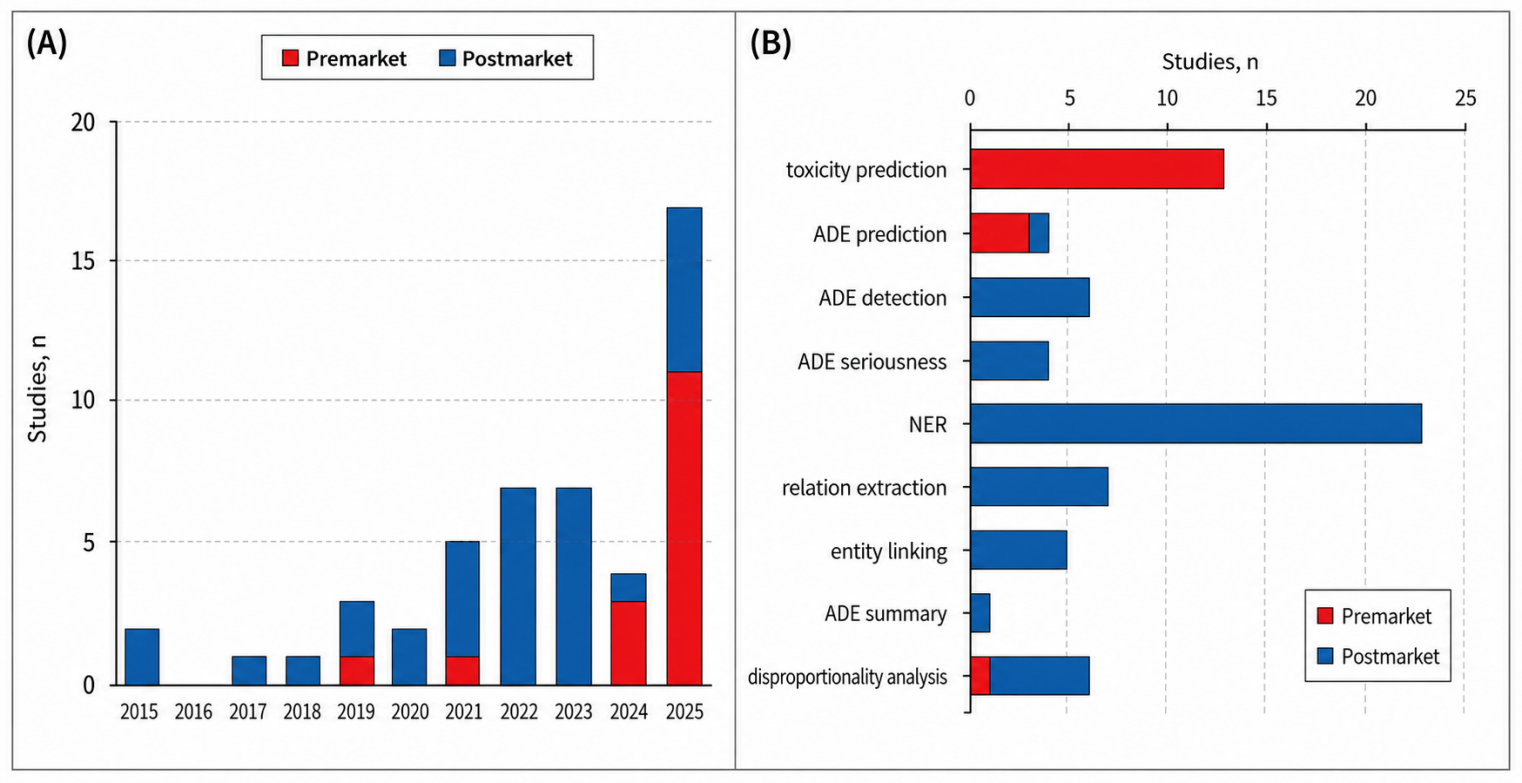
High-level overview of the studies included in the analyses. Trend of artificial intelligence algorithms developed to be used in a premarket or postmarket phase and categorized by (A) application of the algorithm over time, distinguishing premarket applications (red) and postmarket applications (blue) and (B) distribution of studies by task-level application, separating premarket (red) and postmarket (blue) use cases. ADE: adverse drug event; NER: named entity recognition.

This review covers studies published both in scientific journals (n=42) and conferences (n=7). Based on the corresponding author’s affiliation, 11 of 49 papers are located in China, equaling the number in the United States (n=11), 5 in India, and 4 in Korea, followed by 12 other countries. The increased number of studies on this field over the past 10 years (Figure 2A) and the large geographical spread of the included studies highlight the growing global interest in developing AI algorithms based on LMs for Pharma R&D safety risk assessment, as well as the potential for international collaborations in this regard. As we can note from Figure 2B, studies cover a large variety of tasks, including ADE-related information extraction from free text, such as named entity recognition (NER) (n=23) [3,23-31], safety prediction based on molecular structure, such as toxicity prediction (n=13) [32-35], and disproportionality analysis for signal detection (n=6) [28,34,36-39].

**Figure 3.**
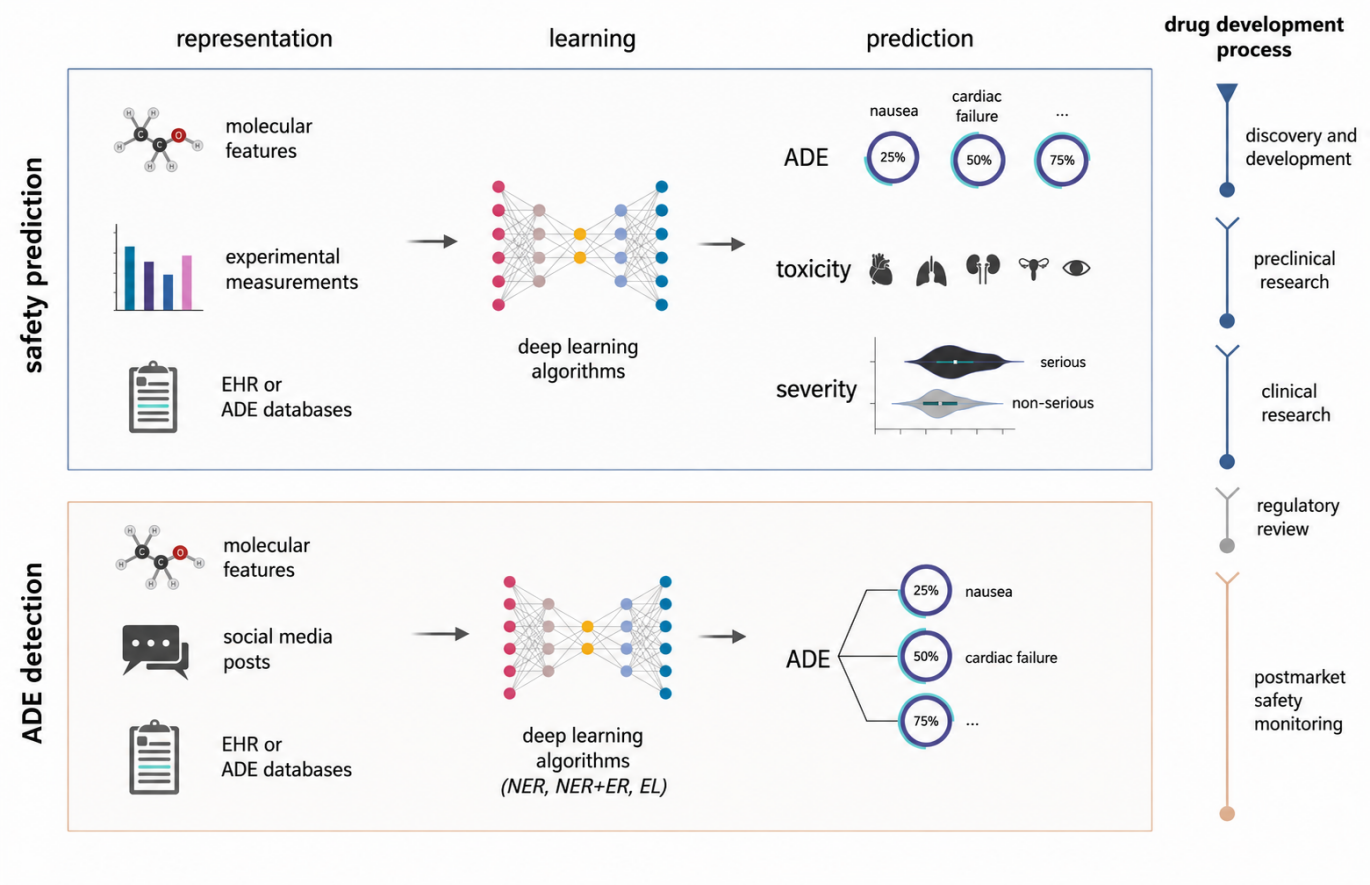
Artificial intelligence applications for safety risk assessment in drug design and development fall into 2 main categories: safety prediction and ADE detection. The artificial intelligence–based analysis process involves three steps: (1) representation: input data, such as chemical compounds and free-text descriptions, are encoded as vectors, heavily supported by learning models. (2) Learning: models are developed to infer safety risks from these data. (3) Prediction: various safety risks are predicted or detected, followed by an evaluation of performance metrics. ADE: adverse drug event; EHR: electronic health record; EL: entity linking; ER: entity relation; NER: named entity recognition.

As shown in Figure 3, we can categorize AI applications for safety risk assessment in drug design and development into 2 main groups: safety prediction and ADE detection. These different applications are found across four of the five stages of drug design and development [40]: (1) discovery and development, (2) preclinical research, (3) clinical research, (4) regulatory review, and (5) postmarket safety monitoring. Due to the challenge of specifying the exact stage that the study addresses, for simplicity, we grouped these 5 stages into 2: pharmaceutical research (premarket) and postmarket safety monitoring (postmarket). Premarket encompasses from stage 1 (discovery and development) to stage 3 (clinical research), including clinical trials from phase I to phase III, while the postmarket stage (safety monitoring—stage 5) includes applications related to clinical trials in phase IV and pharmacovigilance. Regulatory review (stage 4) acts as a bridge between the premarket and the postmarket phases and is not covered in this review.

### Safety Prediction

The safety prediction category (top image of Figure 3) encompasses LM-based applications that can predict safety risks before synthesis and preclinical or clinical testing, given a drug or compound formulation. These applications can be used during the discovery and development stage to support screening and during the preclinical and clinical research stages to support safety risk assessment. Prediction studies can be further subdivided into 3 predictive application use cases: toxicity [32,33,41-48], ADEs [23,49,50], and severity [51]. Toxicity prediction methods are often binary classifiers that predict whether a drug or compound will be toxic for an organ, such as drug-induced liver injury prediction [52-55], or regressors that predict toxicity properties, such as skin sensitization scores [56,57]. In ADE prediction, AI methods supported by LMs are designed to predict the occurrence of ADEs, that is, injuries resulting from the use of a drug. These methods are usually multiclass, multilabel classifiers that infer the occurrence of adverse event categories, such as those proposed by the Medical Dictionary for Regulatory Activities (MedDRA) terminology. Conversely, methods for ADE severity prediction are usually binary classifiers that aim to infer the severity of ADEs, such as serious versus nonserious or death versus nondeath events. In terms of phase, these studies (n=16) are concentrated in the premarket pharmaceutical research stage, with the notable exception of the study by Mazuz et al [51], which focuses on predicting drug withdrawal based on safety concerns.

### ADE Detection

The ADE detection category (bottom image of Figure 3) encompasses LM applications that extract ADE-related information from individual documents in a given corpus so that signal detection can be performed. These detection studies can be further subdivided into 2 categories: information extraction, including NER [3,36,58-65], relation extraction (RE) [24,27,62,66-69], entity linking (EL) [28,36,59,61,62], and document classification, including ADE mentioning in documents [37,70-74] and their seriousness [27,39,75]. NER methods are used to identify ADE-related entities, such as drugs, dosage, route of administration, and ADE names, in relevant pharmacovigilance corpora, such as patient forums [26,28-31,59,61,63,76] and social media [30,31,59-61,63,65,70,71,76]. RE methods are often combined with NER methods to identify relationships between ADE-related entities. For example, they can establish whether a drug is associated with an ADE [24,27,62,66,67], while EL methods are used to normalize ADE entities against standard terminologies in the field, such as MedDRA [23,27,28,39,59,60,62]. These tasks ultimately enable the structuring of ADE-related information found in free-text corpora, allowing for further computation of ADE cases for a given drug and the application of signal detection algorithms. Document classification is a simpler task, in which text passages, such as tweets [70,71,74], posts in patient forums [29,61,76], or incident reports [36,37,62,64,75], are classified as containing ADE information or the seriousness of the reported ADE [27,39,75]. Unlike the information extraction category, the goal here is to triage large corpora to reduce the cost of manual processing or enable further automated information extraction. Stage-wise, these studies (n=33) are concentrated in the postmarket safety monitoring stage (Table 3).

**Table 3.** Overview of included studies by application category across the drug development lifecycle.

Application	Studies
Safety prediction	[23,32-35,41-50,77]
ADE^a^ detection	[3,24-31,36-39,51,58-76]

a ADE: adverse drug event.

### For Which Organs and Toxicity Endpoints Are LMs Used for in Safety Analysis?

ADEs can manifest in various organs; yet, most reviewed studies addressed ADE prediction in a general context rather than focusing on specific organ toxicities. This is mainly the case of AI models for ADE detection in the postmarket safety monitoring stage. These models often leverage terminologies such as the MedDRA [78] or the World Health Organization Anatomical Therapeutic Chemical classification system [79] to infer the occurrence of adverse event categories across broad organ systems [80,81]. When it comes to the pharmaceutical research (premarket) stage, we see a shift toward predicting organ-specific toxicities with binary classifiers, which assess whether a compound will be toxic to particular organs [52,55,82-86].

The heart (n=6) [32-34,41,46,48] and liver (n=4) [32-34,46] emerge as the most studied organs for organ-specific ADE prediction (Table 4), likely due to their roles in drug metabolism and systemic effects, respectively. Our screening shows that predicting ADEs in other organs, such as the brain or pancreas, poses a greater challenge due to limited experimental data and the inherent difficulty that comes from the resource-intensive and complex methodologies needed in assessing toxicity for these organs [87,88].

**Table 4.** Organ systems and toxicity endpoints evaluated in the included studies.

Category and subcategory	Studies, n
Organ	
Heart	6
Liver	4
Bone	2
Skin	2
Eye	2
Other	5
Endpoint	
Cardiotoxicity	7
Hepatotoxicity	4
Carcinogenicity	3
Median lethal dose	3
Peptide toxicity	3
Mutagenicity	2
Osteotoxicity	2
Skin reaction	2
Other	7

Of the many toxicity endpoints that different groups have tried to develop models for, the prediction of cardiotoxicity [32,34,48] and drug-induced liver injury [33,46] are particularly well-represented in the literature (Table 4), reflecting their clinical significance and data availability for these toxicity endpoints. Models able to provide such predictions can serve as a filter to identify potentially harmful drugs in the premarket stage and reduce the failure risk in the later drug development stages.

### What Types of LMs Have Been Used to Analyze Safety Risks in Drug Design and Development?

**Figure 4.**
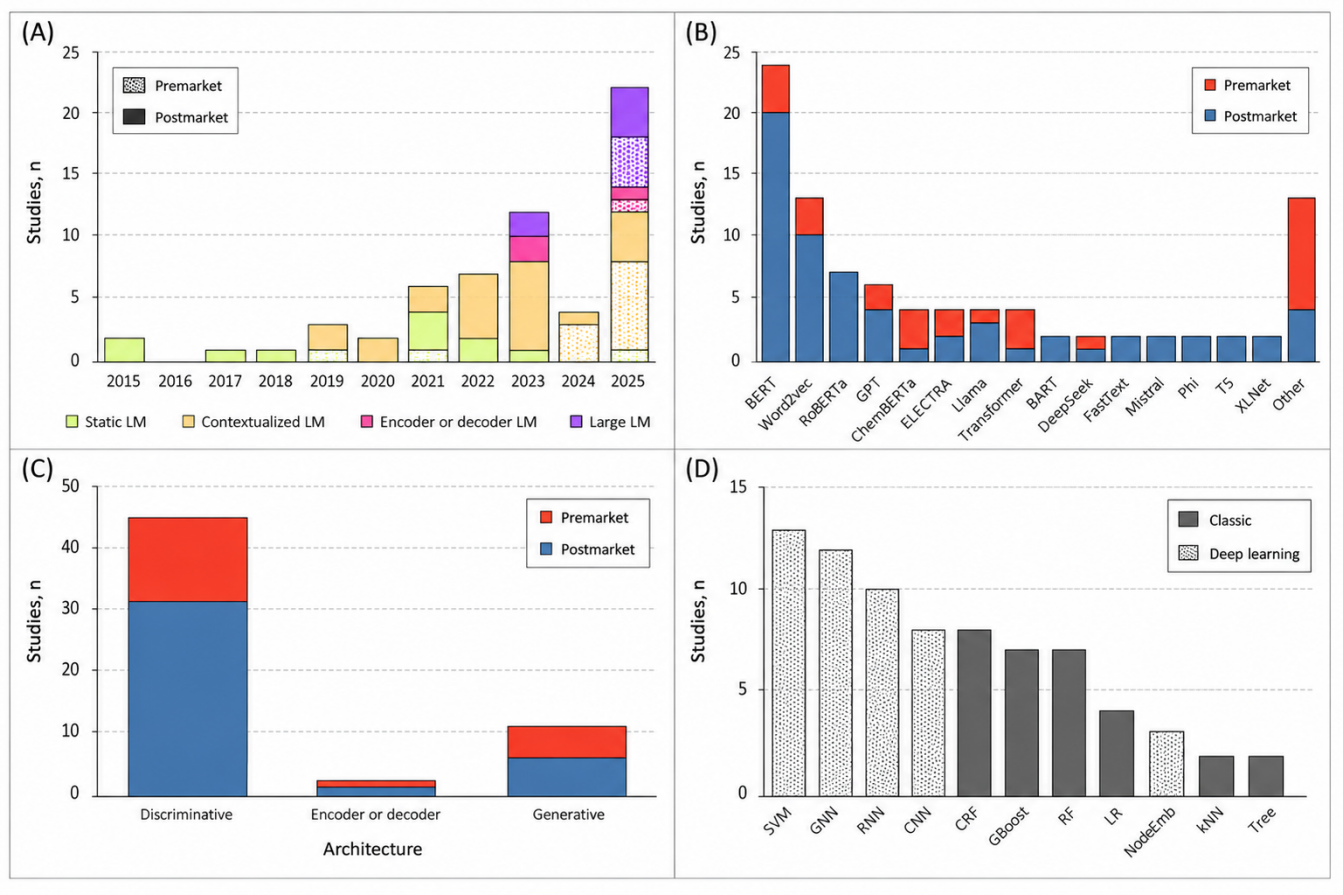
Artificial intelligence algorithms used for safety risk assessment in pharmaceutical research and development. (A) Studies published per year from 2015 to 2025, grouped by LM type: static, contextualized, encoder-decoder, and large. (B) Distribution of studies across different LM architectures. (C) Studies stratified by LM architecture type: discriminative, encoder-decoder, and generative. (D) Classical machine learning and deep learning approaches. BART: bidirectional and auto-regressive transformer; BERT: bidirectional encoder representations from transformers; CNN: convolutional neural network; CRF: conditional random field; ELECTRA: efficiently learning an encoder that classifies token replacements accurately; GBoost: (extreme) gradient boosting; GNN: graph neural network; kNN: k-nearest neighbors; LM: language model; LR: logistic regression; NodeEmb: node embedding; RF: random forest; RNN: recurrent neural network; SVM: support vector machine; Tree: decision tree.

Assessment of LM use over time reveals a clear evolution in methodological choices (Figure 4A). Until 2018, all studies relied exclusively on static LMs [29,65,66,71]. Between 2019 and 2022, a more balanced use of static and contextualized LMs was observed, reflecting a transitional phase toward context-aware representations [3,36,37,49,50,58-61,64]. From 2023 onward, more advanced architectures emerged, with the introduction of encoder-decoder models and LLMs [23,25,30,31,47,51,63], which rapidly gained prominence, accounting for approximately half of the studies included by 2025. Notably, no publication from 2024 used encoder-decoder models or LLMs. This pattern may be because most of the papers published in 2024 that we included in our review [41,42,46] were preclinical studies, which primarily rely on discriminative models rather than more recent generative LMs. However, stochastic variation in publication trends cannot be excluded.

Further analysis of the LMs used across the included studies shows that the majority relied on the bidirectional encoder representations from transformers (BERT) architecture (n=24; Figure 4B) [3,26-28,41,51,59,69,75,77]. This predominance is consistent with the architectural design of BERT as an encoder-only, bidirectional model, which is particularly well-suited for generating high-quality contextualized embeddings for downstream tasks such as classification, similarity analysis, and information retrieval. In contrast, large generative models such as GPT-4 or LLaMA are primarily optimized for autoregressive text generation rather than embedding extraction, making BERT-based models generally more precise and computationally efficient for representation learning purposes. The word2vec model (n=13) was the second most frequently used [36,43,49,50,61,62,64-66,71], followed by more recent approaches, such as RoBERTa (n=7) [26,31,59,70,72,73,75] and GPT (n=6) [30,31,48,63,73,76] architectures. A substantial proportion of studies using these models was conducted for safety risk assessment in postmarket settings. This predominance of postmarket applications can be explained by the fact that the tasks most related to this phase, that is, information extraction and document classification (Figure 2B), rely extensively on natural language processing. In contrast, the use of LMs, such as ChemBERTa, especially for molecular representations in premarket studies, is a more recent adaptation of language modeling to other data modalities.

If we look at the model architectures across the included studies (Figure 4C), we see a clear predominance of discriminative approaches (n=44), that is, LMs that focus on learning the boundaries between different tokens in a corpus, such as BERT [3,41,51,69,75], word2vec [36,43,49,62,64], and XLNet [26,31]. These models are used for tasks such as NER and RE, as well as toxicity and ADE prediction [23,24,49,50,66,67,70-73]. Discriminative models, often implemented as fine-tuned, encoder-based transformer architectures, are particularly well-suited to these objectives, as they are optimized for classification and sequence labeling tasks that rely on well-defined input-output mappings. In contrast, studies leveraging generative LMs, that is, models that generate text by predicting the next token based on preceding context, focus on NER [25,30,31,63,73,76] (n=6), benefiting from the zero-shot learning (ie, without annotated data) capabilities of those models. As seen from Figure 4A, the use of these models has significantly increased in the last year of the survey, benefiting from the recent progress made in LLMs.

LLM-based models are often used in combination with classical ML (n=4) [28,29,66,68] and deep learning (DL) models (n=31) [32,42-44,50,62,64,70,71,76]. Among the ML and DL models used in parallel or in combination with LLMs, support vector machines (SVMs) are the most prevalent (n=13) [28,29,33,41,44,47,48,50,68,71], followed by graph neural networks (GNNs; n=12) [32,33,35,41-43,47,48,51,70,76], then recurrent neural networks (RNNs; n=10) [3,35,43,47,58,60,62,64,65], with convolutional neural networks (CNNs; n=8) [3,35,43,46,47,60,71,74] used at a comparable frequency to conditional random fields (CRFs; n=8) [3,28,29,58,62,64-66]. Overall, SVM and CRF are the most used among classical ML approaches, whereas GNNs, RNNs, and CNNs are predominant among DL methods. In addition, across the reviewed studies, GNNs are primarily used for molecular representations [32,33,35,41-43,47,48,51], whereas SVMs, RNNs, CNNs, and CRFs are mainly used for both molecular representation [33,35,41-44,46-48,50] and clinical text data [3,58,66,68].

### What Are the Data Sources and Metrics Used for Training and Evaluating LM Methods for Safety Assessment in Pharma R&D?

AI-based safety assessment studies in Pharma R&D leverage diverse structured and unstructured data sources for safety prediction and ADE detection (Table 5). Premarket studies focusing on molecular representations primarily rely on curated chemical and pharmacological datasets, with SIDER (n=6) [33,41,44,48-50] and ClinTox (n=3) [33,41,45] being the most frequently used resources, reflecting their central role in modeling toxicity and drug-ADE associations. Knowledge bases such as DrugBank, ChEMBL, and PubChem further complement these analyses by providing chemical and biological context.

**Table 5.** Main datasets used by dataset type and study data focus.

Dataset	Dataset type	Study data focus					All
		Molecular representation	Social media	Clinical text	Scientific literature	Incident reports	
SIDER [29,33,39,41,44,48-50]	ADE^a^-specific knowledge resources	6	2	0	0	0	8
ClinTox [33,41,45]	ADE-specific knowledge resources	3	0	0	0	0	3
ATSE [35,43]	ADE-specific knowledge resources	2	0	0	0	0	2
CTD [44,48]	ADE-specific knowledge resources	2	0	0	0	0	2
DILI^b^ [33,34]	ADE-specific knowledge resources	2	0	0	0	0	2
ToxinPred2 [35,43]	ADE-specific knowledge resources	2	0	0	0	0	2
CT-ADE [25]	ADE-specific knowledge resources	1	0	0	0	0	1
SMM4H (Twitter) [25,26,31,59,60,63,70,76]	Social media–annotated datasets	0	8	0	0	0	8
CADEC (patient forum) [26,31,59,63,76]	Social media–annotated datasets	0	5	0	0	0	5
Twitter [28,29,70,74]	Social media–annotated datasets	0	4	0	0	0	4
Patient forum [28,30,61]	Social media–annotated datasets	0	3	0	0	0	3
ADHD (Twitter) [65,71]	Social media–annotated datasets	0	2	0	0	0	2
PsyTAR (patient forum) [59,76]	Social media–annotated datasets	0	2	0	0	0	2
Reddit [39]	Social media–annotated datasets	0	1	0	0	1	2
DailyStrength (patient forum) [29]	Social media–annotated datasets	0	1	0	0	0	1
EHR (private) [38,66-68,72]	Annotated clinical reports	0	0	5	0	0	5
MADE (EHR) [24,58]	Annotated clinical reports	0	0	2	0	0	2
n2c2 (EHR) [3,24]	Annotated clinical reports	0	0	2	0	0	2
ClinicalTrials.gov [23,45]	Annotated scientific literature	2	0	0	1	0	3
PubMed or MEDLINE [27,50,69,70]	Annotated scientific literature	1	1	0	2	0	4
TAC [59,76]	Annotated scientific literature	0	2	0	0	0	2
ADE-corpus-v2 (PubMed) [27,73]	Annotated scientific literature	0	0	1	2	0	3
FAERS^c^ [34,36,39,44,48,61]	Incident report systems	3	2	0	0	2	7
EU-ADR [38,49]	Incident report systems	1	0	1	0	0	2
ANSM [75]	Incident report systems	0	0	0	0	1	1
FDA [37]	Incident report systems	0	0	0	0	1	1
Health Canada [37]	Incident report systems	0	0	0	0	1	1
Jiangsu ADR Mon. Center [64]	Incident report systems	0	0	0	0	1	1
KAERS [62]	Incident report systems	0	0	0	0	1	1
DrugBank [23,34,41,44,48,50,51,69,76]	Knowledge base, biomedical terminologies, and databases	7	1	0	2	0	10
ChEMBL [23,27,42,45,46,51]	Knowledge base, biomedical terminologies, and databases	5	0	0	2	0	7
PubChem [23,48,50]	Knowledge base, biomedical terminologies, and databases	3	0	0	1	0	4
UniProt [35,43]	Knowledge base, biomedical terminologies, and databases	2	0	0	0	0	2
ZINC [45,46]	Knowledge base, biomedical terminologies, and databases	2	0	0	0	0	2
MedDRA^d^ [23,27,28,39,59,60,62]	Knowledge base, biomedical terminologies, and databases	1	4	0	2	2	9
OMOP [36,38,49]	Knowledge base, biomedical terminologies, and databases	1	0	1	0	1	3
UMLS [29,38,61]	Knowledge base, biomedical terminologies, and databases	0	2	1	0	0	3
ATC^e^ [28,76]	Knowledge base, biomedical terminologies, and databases	0	2	0	0	0	2

a ADE: adverse drug event.

b DILI: drug-induced liver injury.

c FAERS: FDA Adverse Event Reporting System Database.

d MedDRA: Medical Dictionary for Regulatory Activities.

e ATC: Anatomical Therapeutic Chemical.

Postmarket safety monitoring predominantly exploits real-world data, including social media, clinical text, and reporting systems. Twitter-based datasets from SMM4H (n=8) [25,26,31,59,60,63,70,76] and CADEC (n=5) [26,31,59,63,76] are widely used to capture patient-reported ADEs and enable near real-time signal detection. Clinical text-based studies mainly rely on annotated EHRs (n=5) [38,66-68,72], as well as MADE and n2c2 datasets, to support supervised ADE extraction. Across all data focuses, scientific literature sources (eg, ClinicalTrials.gov and PubMed or MEDLINE) [23,27,45,50,69,70] and spontaneous reporting systems such as the FDA Adverse Event Reporting System Database (FAERS) [34,36,39,44,48,61] provide complementary evidence for large-scale pharmacovigilance analyses.

**Figure 5.**
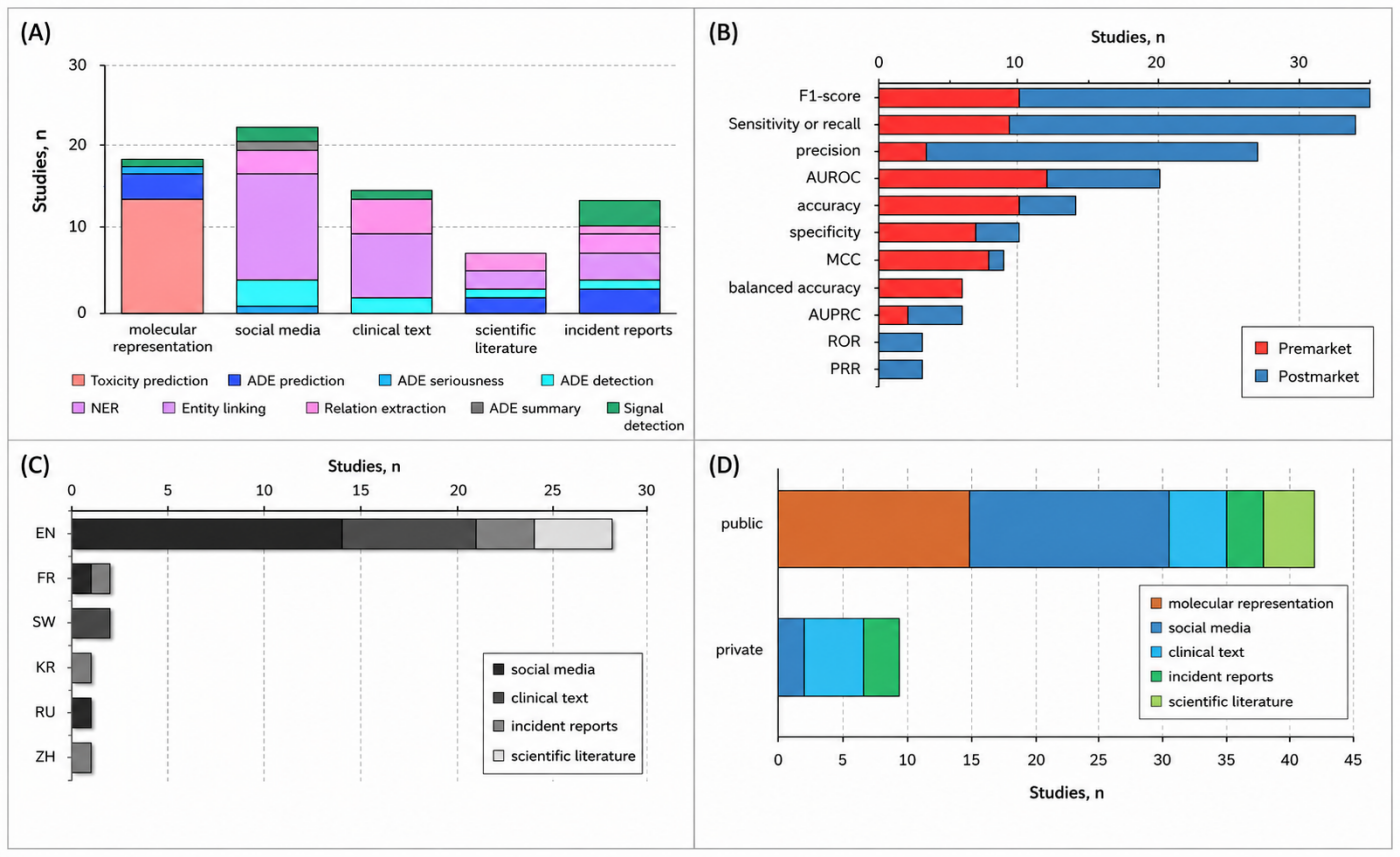
Overview of data sources and evaluation metrics used for training and validation, including data focus by task type, evaluation metrics, dataset language, and dataset accessibility. (**A**) Number of studies by data focus category stratified by task type. (**B**) Number of studies by evaluation metrics. (**C**) Distribution of studies by dataset language. (**D**) Number of studies by dataset accessibility: public versus private. ADE: adverse drug event; AUPRC: area under the precision-recall curve; AUROC: area under the receiver operating characteristic curve; EN: English; FR: French; KR: Korean; MCC: Matthews correlation coefficient; NER: named entity recognition; PRR: proportional reporting ratio; ROR: reporting odds ratio; RU: Russian; ZH: Chinese.

As shown in Figure 5A, distinct methodological trends can be observed with respect to the targeted tasks across the different data focus categories, namely, molecular representation, social media, clinical text, scientific literature, and incident reports. Studies relying on molecular representations predominantly focus on toxicity prediction [32-35,41-48,77], reflecting their emphasis on molecular structure features for safety risk assessment. In contrast, studies leveraging social media, clinical text, scientific literature, and incident reports primarily address NER [3,36,58-65], as these data sources consist largely of unstructured text for which entity identification is a foundational step. Within these text-based domains, social media–based studies also place a strong emphasis on EL, aiming to normalize patient-reported mentions of drugs and ADEs to standardized vocabularies [28,59,61]. Clinical text-focused studies, on the other hand, more frequently target RE as the additional task [24,66-68], seeking to identify explicit associations between drugs and ADEs within clinical narratives. Studies based on incident reports exhibit a broader task spectrum, commonly integrating NER, EL, and RE [36,62,64], in addition to document classification [37] and signal detection [36,37,39], to facilitate triage and fully exploit structured reporting formats for pharmacovigilance signal detection, enabling a comprehensive pharmacovigilance analysis.

With respect to the evaluation metrics (Figure 5B) used, clear differences emerge between premarket and postmarket evaluation practices. Metrics such as the *F*_1_-score, sensitivity (recall), and precision are most frequently reported overall, largely due to their extensive use in postmarket studies [3,27,29,58-60,62,63,69,74]. In contrast, evaluation metrics, including the area under the receiver operating characteristic curve (AUROC), Matthews correlation coefficient, accuracy, specificity, and balanced accuracy, are predominantly used to assess premarket studies [32,33,41-45,48,49,77]. Certain metrics, notably the reporting odds ratio and proportional reporting ratio, are exclusively used in postmarket settings for statistical signal detection [28,34,36-39].

Most of the reviewed papers use English datasets (n=28) [3,23,36,49,58-61,63,70] and publicly available data (n=38) [3,23,41,46-49,58,60,73] (Figure 5D), reflecting the widespread use of open premarketing chemical and postmarketing pharmacological databases in this domain [34,35,48,51,60,61,63,65,71,77]. A minority of papers analyze other European languages, such as French (n=2) [28,75] and Swedish (n=2) [66,67], and Asian languages, such as Korean (n=1) [62] and Chinese (n=1) [64]. In contrast, studies focusing on clinical text are proportionally more likely to rely on private data sources [38,66-68,72], highlighting the restricted access associated with clinical records and institutional electronic health data.

Regarding the features used for different tasks (Table 6), word embeddings and molecular embeddings are the most used. Word embeddings are predominantly used for NER (n=23) [24,25,31,38,63-67,76] but are also applied across most other tasks [37,39,50,68-72,74,75], except for toxicity prediction. In this case, LMs are primarily for feature engineering, converting natural language symbols into dense representations. Toxicity prediction tasks, which are mainly addressed in premarket studies, rely primarily on molecular embedding features (n=10) [32-35,41-43,45,46,48], derived using LMs (molecular or protein LMs to be specific). N-gram features rank third in terms of use frequency and are used to a lesser extent than embeddings, often as complementary features. As with word embeddings, n-grams are not used for toxicity prediction, further highlighting the task’s reliance on structured molecular representations rather than textual features.

**Table 6.** Main features used in the analyzed studies across the different tasks.

Features	NER^a^	Toxicity prediction	ADE^b^ detection	RE^c^	Signal detection	ADE seriousness	EL^d^	ADE prediction	ADE summary
Word embedding	23	0	6	7	5	3	5	2	1
molecular embedding	0	10	0	0	1	1	0	1	0
n-gram	4	0	2	2	2	0	2	0	0
TF-IDF^e^	0	0	3	1	0	1	0	1	0
Molecular property	0	3	0	0	0	1	0	1	0
Lexicon	1	0	2	1	0	0	0	0	0
Molecular fingerprint	0	4	0	0	0	0	0	0	0
Categorical	0	0	0	0	0	1	0	1	0
Molecular descriptor	0	2	0	0	0	0	0	0	0
Node embedding	0	0	0	0	1	1	0	0	0
Protein embedding	0	2	0	0	0	0	0	0	0
Sentence embedding	0	0	1	0	1	0	0	0	0

a NER: named entity recognition.

b ADE: adverse drug event.

c RE: relation extraction.

d EL: entity linking.

e TF-IDF: term frequency-inverse document frequency.

### What Are the Current Limitations of LM Approaches for AI-Based ADE Analysis?

Despite rapid methodological progress, the reviewed literature reveals that there are several persistent limitations that constrain the reliability, generalizability, and practical utility of LM-based methods for safety prediction and detection across the drug development lifecycle.

Many studies rely on English-language corpora [3,49,58,60], spanning social media benchmarks, patient forums, and biomedical literature, as well as many publicly available ADE corpora used in modeling pipelines. This creates a bias, whereby models trained and evaluated primarily on English might underperform when deployed on non-English narratives, where lexical variation or different naming conventions could alter information extraction performance. Beyond language, geographic bias [32,36,61,64] could emerge because prescribing patterns or drug availability vary across health care systems. Models trained on US data sources may not be applicable in other jurisdictions without adaptation.

Although many postmarket studies extract ADE mentions or compute signals from real-world sources, none validate downstream findings against established postmarketing evidence. For example, several studies have extracted and normalized ADE from social media [60,63,65]. However, none of them report how these findings compare with ADEs validated in phase IV clinical trials. Without robust external validation, it remains difficult to quantify the proportion of signals representing actionable findings rather than noise. ADE detection in postmarket settings is often framed as information extraction, primarily NER [30,31,73] and RE [24,27,68], to structure what is stated in narratives. The goal of these systems is not to establish biomedical causality. Instead, they aim to identify causal attributions as expressed by the author, whether clinician or patient, and to make that information usable for downstream pharmacovigilance workflows. However, translating extracted attributions into actionable safety assessments requires further analysis. Real-world safety evaluation must account for factors such as confounding by indication and differences in target populations. This is essential because the likelihood and severity of ADEs are context-dependent and can vary with determinants such as underlying disease, dose, and treatment duration. This contextuality also highlights a key limitation of many premarket resources and toxicity prediction pipelines. These resources and pipelines often rely on decontextualized representations of compounds and therefore miss patient- and regimen-specific determinants that shape how adverse outcomes manifest in practice.

Across studies, evaluation most commonly centers on *F*_1_-score [24,28,48], precision or recall [34,74,75], and AUROC [33,38,48]. In premarket safety prediction studies, AUROC and accuracy are predominant. While these are useful metrics, they should be interpreted with caution in the presence of class imbalance, which is common in toxicity and ADE datasets. In such settings, AUROC or accuracy may mask poor performance on rare but safety-critical events and should therefore be reported alongside more robust metrics such as balanced accuracy, Matthews correlation coefficient, and *F*_1_-score. Moreover, calibration is rarely assessed, despite being essential when model outputs are used to rank safety risks or trigger alerts. A model can achieve strong discrimination while producing poorly calibrated probabilities, which can lead to inappropriate decision thresholds and misinterpretation of predicted risk. In addition, the literature remains skewed toward postmarket detection, with comparatively few LM applications that integrate into early development decisions. This imbalance restricts the ability of LM methods to support proactive risk mitigation, where earlier detection could reduce attrition and patient harm.

## Discussion

### Principal Findings

AI, and LMs in particular, is increasingly influencing how ADEs are anticipated and monitored throughout the drug development lifecycle. The recent acceleration in this field is not simply a result of wider adoption of AI. It also reflects a methodological consolidation where LMs provide a common framework for learning from molecular structure [41-43] and human-generated text [3,58,63]. By mapping LM applications from premarket pharmaceutical research through postmarket safety monitoring, this scoping review captures an evolution from static embedding methods [29,65,66] toward contextualized transformer encoders [45,70,72] and, more recently, LLMs [25,30,63].

Across the 49 studies included in this review, which were published between 2015 and October 15, 2025, LM-based applications are more strongly represented in postmarket ADE detection (n=33) than in premarket safety prediction (n=16). Postmarket studies typically implement pharmacovigilance as information extraction tasks, including NER, RE, EL [59,61,66], and document-level classification [39,51,72,74], tasks for which LMs are naturally designed. In contrast, premarket studies most often frame safety as a predictive modeling problem over compound representations, in which LMs act as feature learners that enable toxicity prediction [32,33,46] before synthesis, extensive preclinical testing, or clinical evaluation. Taken together, these 2 aspects illustrate how LMs function as a bridging technology throughout the process by standardizing representation learning across data modalities and enabling safety assessment within a unified methodological family.

With respect to the safety outcomes addressed, organ-specific prediction remains focused on a limited set of endpoints that are both clinically relevant and well supported by the available training data. Heart and liver toxicities predominate among organ-targeted studies, and accordingly, cardiotoxicity and hepatotoxicity emerge as the most modeled endpoints [32,33,41]. Conversely, less frequently modeled organs, such as the brain [44,48], experience a scarcity of high-quality labeled datasets and the experimental complexity required to generate ground truth for these tissues. As a result, methodological progress is currently strongest, where data availability is favorable, while important gaps persist for rarer, complex, or difficult-to-measure toxicities.

### Comparison to Prior Work

The included literature shows a clear methodological progression from static representations in earlier years to contextualized encoders and, more recently, to the recent emergence of LLMs. Despite increased attention to generative systems, most of the included studies remain centered on discriminative, encoder-based architectures, aligning with the predominance of supervised extraction and classification tasks. BERT-family models are the most frequently used contextualized architectures [32,45,76], while word2vec remains common [36,43,61], often within hybrid pipelines that combine learned embeddings with classical ML or DL classifiers [50,66,71]. The continuing dominance of discriminative approaches is consistent with their comparatively lower computational requirements and stable supervised training behavior, as well as the availability of annotated datasets suited for supervised learning [50,65,72]. Furthermore, the pharmaceutical industry’s regulatory framework favors deterministic reliability over generative flexibility. While LLMs offer powerful zero-shot capabilities, their propensity for hallucination poses a safety risk in pharmacovigilance, where a fabricated ADE signal is as dangerous as a missed one. The future dominance of LLMs will likely depend less on their generative fluency and more on the development of guardrails and grounding mechanisms that can satisfy regulatory rigor.

At the same time, the increasing appearance of LLMs, particularly in the most recent portion of the review period, suggests a shift toward approaches that can exploit zero-shot or few-shot capabilities, which could reduce dependence on costly annotation. In the reviewed evidence, however, this generative turn is most represented in postmarket extraction settings rather than spanning the full drug development lifecycle uniformly.

### Future Directions

The data ecosystems underlying pre- and postmarket applications differ systematically. Premarket prediction studies largely rely on curated chemical and toxicity resources (eg, SIDER, ClinTox, and CTD) [44,45,49] and are frequently complemented by established knowledge bases providing chemical and biological context (eg, DrugBank, ChEMBL, and PubChem) [44,45,50]. Postmarket detection studies predominantly leverage real-world narrative sources, including social media benchmarks (eg, SMM4H and CADEC) [31,63,76] and clinical text resources (eg, private EHR datasets, MADE, and n2c2) [3,24,72]. These differences in the data are mirrored in the evaluation practices, with postmarket studies typically reporting precision, recall, and *F*_1_-scores [58,60,62], whereas premarket studies more frequently emphasize AUROC and accuracy [32,34,77]. While such metric choices are conventional for the respective task families, they complicate comparisons across pipeline stages and can obscure safety-relevant weaknesses. High entity-level *F*_1_, for example, does not necessarily translate into reliable pharmacovigilance decisions unless downstream signal utility is demonstrated. Similarly, AUROC and accuracy can remain favorable under class imbalance, potentially masking poor performance on rare but clinically significant events. Consequently, the field experiences a metric-utility misalignment. In safety surveillance, the cost of a false negative (missing a fatal ADE) vastly outweighs the cost of a false positive (unnecessary review). Yet, many reviewed studies optimize for balanced metrics like *F*_1_-score rather than prioritizing high-sensitivity configurations (recall>95%) that act as effective safety nets. Future benchmarks must penalize missed (serious) signals more heavily to reflect the operational realities of drug safety.

Beyond evaluation metrics, the reviewed literature reveals several recurring limitations. Linguistic and geographic bias in training data remains common. Downstream validation is often limited, and extracted ADE information is rarely leveraged in deeper analyses, while calibration is also infrequently assessed. Together, these issues constrain the generalizability and practical utility of LM-based pharmacovigilance systems. LM-based methods are appealing because they can support safety analyses across the drug development lifecycle while preserving existing practices. However, their value proposition depends on moving beyond isolated tasks toward demonstrable gains in workflow efficiency and patient safety.

Taken together, the evidence indicates that LM-based methods have evolved from representation learning into a diverse toolkit supporting both premarket safety prediction and postmarket ADE detection. While the field remains anchored in discriminative transformers and supervised training, generative LLMs are emerging as a complementary paradigm that could accelerate adaptation thanks to their zero-shot capabilities. Future progress is likely to depend strongly on robust generalizability and downstream validation. Moreover, there is a clear need for greater methodological convergence between chemical LMs and text-based LMs, particularly through multimodal approaches that incorporate chemical-, patient-, and regimen-level information. Currently, the bridge provided by LMs is methodological rather than functional; premarket models do not learn from postmarket text, and vice versa. A true paradigm shift will occur only when multimodal architectures are trained jointly on molecular structures and safety-related narratives. This would allow a model to analyze a chemical structure and directly generate its potential postmarket safety profile, effectively closing the feedback loop that currently takes years to traverse [23]. In the context of safety prediction, such efforts would help bridge the gap between decontextualized, compound-centric predictions and the context-dependent determinants of ADE risk in real-world scenarios. This suggests that the performance ceiling in toxicity prediction is not algorithmic, but conceptual. By modeling molecules as isolated static entities, current LMs ignore the physiological context (eg, metabolism and genetics) that defines toxicity. Future breakthroughs will require moving from molecule-centric LMs to interaction-centric systems that embed compounds within virtual biological environments.

### Strengths and Limitations

This review has several limitations. First, the scope was deliberately narrowed to studies that explicitly use LM techniques. While this keeps the research questions we posed tractable, it inevitably underrepresents the large body of preclinical safety work that still relies on nonlinguistic DL (eg, pure graph or image models) and may therefore give the impression that the use of LMs is more mature than it really is [18], or that it is the only approach to apply AI for drug safety. Another limitation lies in the fact that almost all of the papers ultimately included analyze English corpora, with only a handful making use of non-English corpora. This linguistic bias, reinforced by the exclusion of non-English papers, already criticized in earlier pharmacovigilance reviews [16], limits the study’s external validity in markets where social media posts in languages, such as Spanish, Portuguese, Hindi, and Arabic, dominate. This mirrors the selection bias that Chekroud et al [89] have highlighted for efficacy prediction using clinical trials and probably inflates the apparent dominance of English-language, Twitter-based, postmarketing studies. From a methodological point of view, our search strings were compiled using high-level terms (eg, “language model”) and will have missed papers that mention only specific algorithms (eg, “ELMo” and “BERT”) without the umbrella “LM” label.

### Conclusions

While the application of LMs to ADE analysis in pharmaceutical research and development is still in its early stages, this scoping review highlights the field’s rapid maturation and considerable potential. Our findings demonstrate that LMs can be integrated across various stages of the drug development lifecycle, from early toxicity prediction in the premarket phase to large-scale ADE detection in postmarket surveillance.

The reviewed studies indicate that recent advances in contextualized LMs and LLMs have led to meaningful improvements in the extraction, representation, and analysis of safety-related information from both molecular and textual data sources. As these methodologies continue to evolve, they show great promise in enhancing drug safety assessment, improving the efficiency of pharmacovigilance systems, and ultimately reducing preventable patient harm and health care costs.

Looking ahead, broader adoption of LLM-based approaches will depend on continued progress in model validation, interpretability, and integration into real-world regulatory and clinical workflows. With sustained methodological refinement and closer alignment with pharmacovigilance practice, LMs are well-positioned to play a transformative role in future drug safety monitoring and decision-making, thereby fostering greater trust and acceptance in therapeutic and regulatory settings.

## Supplementary material

10.2196/77732 Checklist 1 PRISMA-ScR checklist.

## Abbreviations

ADE: adverse drug event
AI: artificial intelligence
AUROC: area under the receiver operating characteristic curve
BERT: bidirectional encoder representations from transformer
CHARMS: Critical Appraisal and Data Extraction for Systematic Reviews of Prediction Modeling Studies
CNN: convolutional neural network
CRF: conditional random field
DL: deep learning
EHR: electronic health record
EL: entity linking
GNN: graph neural network
LLM: large language model
LM: language model
MedDRA: Medical Dictionary for Regulatory Activities
ML: machine learning
NER: named entity recognition
Pharma R&D: pharmaceutical research and development
PRISMA-ScR: Preferred Reporting Items for Systematic Reviews and Meta-Analyses Extension for Scoping Reviews
RE: relation extraction
RNN: recurrent neural network
SVM: support vector machine
